# Addressing differences in cancer: a framework for synergistic programming in cancer prevention and control

**DOI:** 10.21203/rs.3.rs-4046415/v1

**Published:** 2024-03-18

**Authors:** Ciaran M. Fairman, Christine M. Kava, K. Beima-Sofie, M. Sakhuja, M. Masud, E. Dias, J. Sheng, J. Gorzelitz, A. Morshed, B. B. Green, M. B. Skiba, P. Madhivanan, N. Parthasarathy, R. Hirschey, M.W. Vander Weg, J Hebert

**Affiliations:** University of South Carolina; Centers for Disease Control and Prevention; University of Washington; University of South Carolina; University of Washington; UTHealth Houston School of Public Health; University of Wisconsin-Madison; University of Iowa; Emory University; Kaiser Permanente Washington Health Research Institute; University of Arizona; University of Arizona; UTHealth Houston School of Public Health; University of North Carolina and Lineberger Comprehensive Cancer Center; University of Iowa; University of South Carolina

**Keywords:** cancer prevention and control, health disparities, frameworks, breast cancer, health equity

## Abstract

**Background:**

Cancer remains a leading cause of death worldwide and continues to disproportionately impact certain populations. Several frameworks have been developed that illustrate the multiple determinants of cancer. Expanding upon the work of others, we present an applied framework for cancer prevention and control designed to help clinicians, as well as public health practitioners and researchers, better address differences in cancer outcomes.

**Methods:**

The framework was developed by the Cancer Prevention and Control Research Network’s Health Behaviors Workgroup. An initial framework draft was developed based on workgroup discussion, public health theory, and rapid literature review on the determinants of cancer. The framework was refined through interviews and focus groups with Federally Qualified Health Center providers (*n*=2) and cancer patients (*n*=2); participants were asked to provide feedback on the framework’s causal pathways, completeness, and applicability to their work and personal life.

**Results:**

The framework provides an overview of the relationships between sociodemographic inequalities, social and structural determinants, and key risk factors associated with cancer diagnosis, survivorship, and cancer morbidity and mortality across the lifespan. The framework emphasizes how health-risk behaviors like cigarette smoking interact with psychological, psychosocial, biological, and psychosocial risk factors, as well as healthcare-related behavior and other chronic diseases. Importantly, the framework emphasizes addressing social and structural determinants that influence health behaviors to reduce the burden of cancer and improve health equity. Aligned with previous theory, our framework underscores the importance of addressing co-occurring risk factors and disease states, understanding the complex relationships between factors that influence cancer, and assessing how multiple forms of inequality or disadvantage intersect to increase cancer risk across the lifespan.

**Conclusions:**

This paper presents an applied framework for cancer prevention and control to address cancer differences. Because the framework highlights determinants and factors that influence cancer risk at multiple levels, it can be used to inform the development, implementation, and evaluation of interventions to address cancer morbidity and mortality.

## Background

Cancer remains a leading cause of death worldwide [1]. In the United States, approximately 21% of all annual deaths are due to cancer, a proportion that is second only to ischemic heart disease [2]. While cancer-related mortality rates have consistently declined since 1991 [2], over 1.7 million new invasive cancer cases were reported in the United States in 2019 [3]. Improved survival, coupled with a growing population, results in over 18 people million living with cancer [4]. Genetic factors such as inherited mutations play an important role in only 5%–10% of cancers [5]. In contrast, preventable risk factors such as substance use, poor nutrition, physical inactivity, and obesity contribute significantly to cancer incidence, survival, and mortality [6–9]. These risk factors commonly co-occur and act synergistically to produce more virulent, harder-to-treat cancers and other disease [10, 11].

There is increasing recognition of the role of social and structural determinants of health, which include environmental conditions and “historical, systemic, structural, and political forces” that impact health [12, 13]. Healthy People 2030 categorizes these determinants into five domains: 1) economic stability; 2) education access and quality; 3) healthcare access and quality; 4) neighborhood and built environment; and 5) social and community context [13]. Social and structural determinants—such as access to healthcare, housing discrimination due to race, poverty, and the built environment—predict cancer incidence, progression, and mortality [14–20]. Intermediate outcomes such as psychosocial stress are also associated with cancer [21, 22], which disproportionately affect certain groups [21]. If not adequately addressed, differences in cancer-related outcomes are likely to increase over time [23], even as overall mortality rates decline.

Frameworks and theories can be used to guide the design, implementation, and evaluation of interventions to address health differences. Several frameworks have been developed to help illustrate the multiple determinants that impact cancer. Notably, Alcaraz et al. [23] describe a blueprint for advancing cancer equity that emphasizes structural inequalities and social injustice as the root causes of health-related differences. Those authors [23] present a framework illustrating the relationships between social determinants, risk factors, and cancer-related outcomes, and include recommendations on how to address social determinants to reduce cancer differences.

Expanding upon the framework of Alacaraz et al. [23] and others, we developed a framework that can be used by clinicians in diverse healthcare settings (e.g., primary care providers in Federally Qualified Health Centers [FQHCs]), by public health practitioners (e.g., state and local health departments staff), and by public health researchers to further guide cancer prevention and control research and practice. The framework examines multiple factors that affect cancer diagnosis, survivorship, and cancer morbidity and mortality. Like the National Cancer Institute [24], we define survivorship as the effects of cancer and its treatment on cancer survivors, including physical, psychological, social, economic, and spiritual effects.

Although others have considered the individual and combined impact of the factors described above, our framework focuses more specifically on how such factors interact with and influence health behaviors relevant to cancer prevention and control. Specifically, we emphasize the need to understand how individuals and communities engage in health behaviors, and the determinants of these behaviors, to more effectively address cancer differences. Our framework incorporates constructs from several theories and approaches relevant to addressing health differences in cancer, including syndemic theory [25], intersectionality [26], life-course perspective [27], and systems science [28]. Singer et al. [25] define a syndemic as the “population-level clustering of social and health problems” (p. 942). Syndemic theory provides a framework to understand how diseases, health conditions, and social conditions interact and worsen population health. Importantly, syndemic theory recognizes that these adverse interactions often emerge under conditions of inequality [25].

Our framework also takes into consideration the proposed influence of commercial determinants of health on cancer, which are not well explored or represented in previous frameworks. Commercial determinants, a subset of social and structural determinants [29], are strategies used by the private sector to produce, sell, and promote products and influence choices that have a negative impact on the environment and population health [30, 31]. Corporate activities influence the social and physical environment and demand for commodities, which in turn shape health behaviors and risk for chronic diseases, including cancer [29]. As described by Maani et al. [32], “the consideration of commercial actors is frequently understated, not made explicit, or simply missing in many of the most influential conceptual frameworks addressing the social determinants of health” (p. 662).

While this framework is applicable to many types of cancer, we use breast cancer as an example of how our framework could be applied to address cancer differences. We selected breast cancer for several reasons, including its high prevalence and associated morbidity and mortality [2], the fact that screening routinely occurs in primary care settings [33], and its significant associations with several health behaviors, including alcohol use, physical activity, and overweight/obesity [34, 35]. Further, significant differences in breast cancer incidence and mortality exist [2], and there are limitations to existing research. An example is a lack of research on estrogen receptor- and triple-negative breast cancers, which are more common among African American women [36]. The purpose of this paper is to outline the methods of developing and refining a framework to inform cancer prevention and control research and practice. We provide examples of the framework’s application within the context of breast cancer.

## Methods

The framework was developed by the Cancer Prevention and Control Research Network’s (CPCRN) Health Behaviors workgroup. The CPCRN, a partnership between research centers in the United States [37], is funded by the Centers for Disease Control and Prevention (CDC) and includes collaborators from the National Cancer Institute. The goal of the CPCRN is to bring together voices of community, academic, and public health partners to develop strategies and implement interventions that are community-informed, focus on equity, and improve cancer outcomes [38].

We developed an initial draft of the framework through workgroup discussion about factors contributing to cancer risk. Discussion occurred during monthly workgroup meetings between March and July 2020. The framework was refined through a rapid literature review, with the goal of synthesizing available evidence as a first step to communicating with communities of solution on the framework development [39, 40]. We searched for published literature in multiple databases, including PubMed, Embase, and Google Scholar. Searches were conducted using keywords in five areas: 1) theories and frameworks relevant to cancer health equity, including syndemic theory and intersectionality, 2) social and structural determinants of health, 3) health behaviors and cancer risk, 4) biologic factors and cancer risk, and 5) influences on cancer survivorship.

We prioritized searching for systematic reviews, meta-analyses, and seminal publications. We reviewed 47 publications and performed abstraction using a standardized form to capture information relevant to the main components of our initial framework. Seven members of the workgroup reviewed and discussed the completed abstraction forms to synthesize current evidence. We then revised the framework to further describe determinants and risk factors associated with cancer differences. In addition to the rapid review, we incorporated constructs from the Dover and Belon [41] Health Equity Measurement Framework, the Alcaraz et al. [23] Integrated Conceptual Framework for Understanding and Addressing Social Determinants to Advance Cancer Health Equity, Healthy People 2030’s Social Determinants of Health Framework [13], and the National Institute on Minority Health and Health Disparities Research Framework [42].

To further refine our framework, five workgroup members trained in qualitative interviewing conducted two 60-minute individual interviews with cancer survivors and two 90-minute group interviews with providers from FQHCs (n=2 individuals) and cancer patients (n=2 individuals). We chose to interview providers from FQHCs since these Centers provide care to patients who lack adeqauate health insurance coverage or access to primary care services; many of these patients, including those with lower incomes, experience worse cancer survival [43]. We recruited participants between November 2022 to March 2023 through CPCRN networks and social media (e.g., LinkedIn and Twitter) promotion. Participants provided verbal informed consent to participate and received $100 e-gift cards as an incentive. Interviews were conducted online using Zoom and were audio recorded.

Interviews were facilitated using a discussion guide that included questions for participants on four topics: 1) their direct and indirect experiences with cancer, including the impact of cancer on their community and life, and whom they turn to for cancer-related information, advice, or support; 2) the framework’s causal pathways, including potential changes that could be made based on participants’ understanding of the causes of cancer; 3) the completeness and organization of the framework, including whether certain components needed to be clarified or better defined; and 4) the applicability of the framework to their work and personal life, including how it could be used to better understand the causes of cancer or develop interventions to address differences in cancer outcomes.

The interviews were not professionally transcribed; instead, interviewers took detailed notes during each interview to capture perspectives on the framework and to identify potential adaptations. Workgroup members who were not present during every interview independently reviewed interview audio against interview notes and confirmed interpretation. Among participants, the overall impression of the framework was positive. Participants expressed that the framework adeptly covered a wide array of concepts related to cancer, spanning the periods before, during, and after diagnosis.

**Figure 1:**
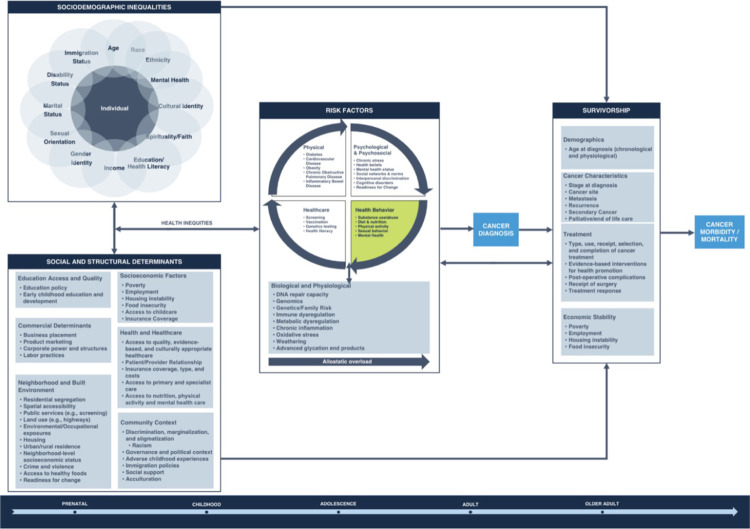
Framework for Cancer Prevention and Control. Sociodemographic inequalities related to individual characteristics (e.g., racism) are bidirectionally associated with social and structural determinants of health. Combined, these factors can create differences in health that can impact more proximal risk factors for cancer, including health behaviors and health conditions. The combined effect of sociodemographic inequalities, social and structural determinants, and risk factors can directly and indirectly impact cancer diagnosis, survivorship, and cancer morbidity and mortality across the lifespan.

Participant feedback was discussed among workgroup members and incorporated into the final framework (Figure 1). For example, participants described the importance of social support for cancer. With this feedback, we added this construct to community context, listed under the social and structural determinants domain of the framework. These modifications enhanced the framework’s comprehensiveness and aligned it more closely with community perspectives and experiences.

## Results

We summarize our final framework below. Many of the framework’s domains (e.g., social and structural determinants) and associated constructs (e.g., features of the neighborhood and built environment) have been well described by previously published frameworks like that of Alcaraz et al. [23]. Given this, we focused our summary on 1) how each domain and its associated constructs influence health behaviors relevant to cancer prevention and control and 2) how key concepts such as syndemics and intersectionality are reflected in our framework. We use breast cancer as an example throughout to illustrate how the framework could be applied by public health and clinical practitioners to address differences in breast cancer outcomes.

Our framework (Figure 1) provides an overview of the relationship between sociodemographic inequities, social and structural determinants, and key risk factors for cancer across the lifespan. Importantly, a primary emphasis of our framework is the relationship between, and impact of, key health behaviors and cancer. It is well established that diet and nutrition, physical activity, certain infections, and substance use and abuse are all explicitly linked to risk of cancer diagnosis [6, 8, 10, 44–46]. These health behaviors interact with key psychological, psychosocial, biological, and physiological risk factors, and such behaviors can also impact healthcare-related behaviors and risk for other chronic diseases such as diabetes and cardiovascular disease [47, 48].

Health behaviors are also intimately related to social and structural determinants of health; ignoring these determinants can result in an unfair attribution of poor diet to behavioral or biological factors rather than differences in access to healthy foods. Consequently, addressing social and structural determinants that influence health behaviors is key to reducing the burden of cancer and improving health equity.

Syndemic theory can be applied to clarify relationships among our framework’s constructs [25]. First, we underscore biological (e.g., family risk) and behavioral (e.g., substance abuse and physical activity) risk factors that co-occur, as visually represented on our framework by the circular arrows in the risk factors domain. In turn, these risk factors can produce biological and physiological changes that create a “wear and tear” on the body (allostatic overload) that can increase risk for cancer diagnosis and negatively impact cancer survivors [49–52]. Second, our framework describes how cancer diagnosis and survivorship vary by multiple characteristics and determinants. For example, certain racial and ethnic populations, including persons who are African American, Hispanic, and Native American, are more likely to experience higher cancer incidence, delayed diagnoses, and poorer survival rates compared to persons who are non-Hispanic White [53, 54]. Also unequally distributed across populations are social and structural factors such as access to healthy foods, access to quality and affordable healthcare for cancer screening, and social support [55]. Consistent with syndemic theory, our framework describes the complex relationships among factors that influence cancer, including how they interact to exacerbate conditions of inequality across the lifespan.

Our framework provides guidance to understand the ways that multiple forms of inequality or disadvantage intersect with one other to create obstacles that often are not considered within conventional ways of thinking about health differences [56]. Social categories are considered to have iterative rather than mutually exclusive effects [56]; this iterative effect is represented in our framework by the overlapping circles for individual characteristics in the sociodemographic inequalities domain. For instance, a person holding the identities of “Black” and “female,” as compared to a White female, may be at greater risk for poor health due to historic economic and social marginalization of Black people. Being in poor health could further contribute to the uptake of health-risk behaviors such as physical inactivity, one risk factor for breast cancer [34].

It is also important to view this framework from a life-course perspective, as the experiences of individuals across different developmental periods can impact cancer outcomes [57, 58]. This perspective is represented on a continuum, from prenatal development to older adulthood, at the bottom of our framework. Our framework recognizes the significant impact of early-life experiences on the likelihood of developing cancer in later years. For example, where high socioeconomic status (SES) is linked with better health outcomes, children born in families with lower SES have more limited resources and experience increased exposure to stressors and health risks that can have long-term effects on breast cancer risk [59].

This disadvantage can build over the life course and lead to accumulated stress [60]. Disadvantage may contribute to unhealthy coping behaviors, such as tobacco use, alcohol drinking, unhealthy eating, and can influence cancer outcomes, including breast cancer, into adulthood [61–63]. Taken collectively, the information above underscores the importance of addressing cancer differences at a systems level rather than focusing on how individual factors contribute to cancer risk in isolation.

## Discussion

Our framework presents relationships among multiple determinants and risk factors that can impact cancer diagnosis, survivorship, and morbidity and mortality at various stages of life [64]. Below, we provide more examples of how the framework can be applied to address differences in cancer outcomes, using breast cancer screening as an example of a key healthcare-related behavior.

### Application

Mammography is associated with early detection and a higher likelihood of survival [65]. Despite screening recommendations from the U.S. Preventative Services Taskforce [66], World Health Organization [67], National Comprehensive Cancer Network [68], and others, some groups have lower screening rates. Our framework posits that social and structural determinants, such as limited access to quality, evidence-based, and culturally appropriate healthcare, increase risk factors that can directly and adversely affect cancer diagnoses and could be targeted to improve screening rates. For example, some studies have found lower rates of breast cancer screening in rural versus urban areas [69, 70]; lower density of primary care providers and radiology facilities in rural areas are noted barriers to screening [70].

Our framework reflects bidirectional relationships between individual-level characteristics and structural determinants, showing that neighborhood context can drive health inequalities and impact cancer risk. Policy- and systems-level efforts such as Medicaid expansion through the Affordable Care Act have been shown to have significant positive impacts on cancer screening, treatment, and health-related outcomes [71]. Yet, our framework shows that these outcomes are driven by interactions between determinants at multiple levels; implementation of one policy or program alone may not be sufficient to improve outcomes.

For example, in one study comparing cancer screening in Appalachian versus non-Appalachian states following a Medicaid coverage expansion, increases in breast cancer screening prevalence were not observed [72]. The authors point to larger structural barriers of cancer screening (e.g., administrative barriers and transportation issues) beyond coverage alone. Previous efforts to increase mammography in low-income populations include mobile mammography clinics and the CDC’s National Breast and Cervical Cancer Early Detection Program [73, 74]. Additional interventions targeting cancer screening and follow-up could use our framework to design strategies that address cancer risk at multiple levels.

Breast cancer screening is a key preventive health behavior, and lack of screening may be associated with other risk factors contributing to health differences. These risk factors do not operate in a vacuum and indeed are highly interrelated. These are a few examples of the differences in access to care and the value of screening practices, which may reflect distrust in healthcare systems, contributing to other risk factors and higher disease burden. Using our framework may aid in understanding the relationships between sociodemographic inequalities, social and structural determinants, and their combined impact on upstream risk factors.

### Recommendations for Intervention

Our framework could inform the development, implementation, and evaluation of interventions to address cancer morbidity and mortality. First and foremost, it is crucial to address the social and structural determinants causing differences in cancer outcomes. Our framework identifies access to quality, evidence-based, and culturally appropriate healthcare as a social and structural determinant. For breast cancer, reducing out-of-pocket costs for screening, using patient navigation services in healthcare settings, and engaging community health workers to connect communities and healthcare systems are strategies recommended by The Community Guide to Preventive Services to reduce healthcare access barriers, improve offering culturally appropriate services, and increase screening rates [75–77]. Similar to Alcaraz et al. [23], clinicians and public health practitioners could conduct focused outreach among groups with lower access to cancer prevention, detection, and treatment interventions to address widening health differences. For example, nurse practitioners within a New Jersey FQHC developed and implemented a quality improvement project that increased mammography rates from 23% to 40% [78].

Our framework illustrates the relationships between social and structural determinants and health behaviors like physical activity. Features of the neighborhood and built environment, including better walkability and greater perceived safety, are associated with higher levels of physical activity [79]. Recommendations to address the living environment offered by Alcaraz et al. [23] include: enhanced surveillance of social factors that contribute to cancer risk and increased engagement of agencies outside of the health sector to address equity. In the example above, public health researchers could collect and incorporate data on neighborhood walkability into existing surveillance systems. At the healthcare level, electronic medical record systems could be adapted for clinicians to capture data on social determinants of physical activity. Collaboration between public health departments and urban planning agencies could support better infrastructure for physical activity [80], which in turn could reduce cancer risk.

Our framework highlights determinants and factors that influence cancer risk at multiple levels, including the policy, community, organizational, interpersonal, and individual levels [81]. Using multi-level interventions to address these influences simultaneously is recommended to reduce health differences [82]. For example, physical activity interventions that incorporate behavior change strategies [83]—tailored to the needs of breast cancer patients and survivors [84, 85] that address environmental and structural barriers—could be tested and implemented by public health researchers and practitioners within communities that have higher breast cancer incidence and mortality rates.

Our framework was informed by several theories and fields of study, including syndemic theory, which provides a useful foundation to understand structural drivers of disease and how multiple disease states interact [25, 86, 87]. For example, Wilson et al. [88] used syndemic theory to explore HIV infection vulnerability among Black and Latino men, highlighting interactions between HIV/AIDS, substance abuse, trauma, incarceration, and poverty. Our framework also highlights interactions between social conditions like poverty and disease, including cancer and other co-morbid conditions. Use of our framework, in combination with health equity theory, could help public health researchers and practitioners identify the most effective and impactful intervention strategies for multiple diseases at multiple levels.

Systems science also recognizes the interconnectedness and complexity of factors that influence disease determinants and outcomes [28]. As fundamental cause theories of health inequities emerge [89], health exposures are embedded over the life course via dynamic processes shaped by networked social structures. Systems science tools help conceptualize these processes and identify areas for interventions that avoid reifying and worsening existing inequalities [90]. These tools could be used by a wide variety of audiences and implementing partners. For example, Wheeler et al. [91] describe how the CPCRN’s Modeling Evidence-Based Intervention Impact workgroup used systems science approaches (e.g., discrete choice survey techniques) to understand the expected impacts of implementing evidence-based interventions for colorectal cancer screening. These approaches could be used by public health researchers to inform the development and implementation of interventions for breast cancer screening, in partnership with healthcare organizations and public health departments.

## Conclusions

This paper presents an applied framework for cancer prevention and control, which describes the relationships among sociodemographic inequalities; social and structural determinants of health; individual risk factors, including health behaviors; and cancer outcomes. To complement currently published frameworks, we focused on the interaction between health behaviors and social and structural determinants of health, while incorporating constructs from multiple theories relevant to health equity, including syndemics. We also sought input from diverse groups (e.g., FQHC providers, cancer patients) during the development process to ensure that the framework would be relevant to key implementing partners in cancer prevention and control. Our framework underscores the importance of addressing social and structural determinants of health to reduce differences in cancer outcomes.

## Data Availability

Data sharing is not applicable to this article as no datasets were generated or analysed during the current study.

## Abbreviations

CPCRN: Cancer Prevention and Control Research Network
CDC: Centers for Disease Control and Prevention
FQHC: Federally Qualified health center
SES: Socioeconomic status
